# Consumption of Antibiotics in Primary Care Setting before and during COVID-19 Pandemic in Republic of Srpska, Bosnia and Herzegovina

**DOI:** 10.3390/antibiotics11101319

**Published:** 2022-09-28

**Authors:** Dragana Sokolović, Dragana Drakul, Bojan Joksimović, Nenad Lalović, Nada Avram, Marija Milić, Dajana Nogo-Živanović, Biljana Mijović

**Affiliations:** 1 Faculty of Medicine Foča, University of East Sarajevo, Studentska 5, 73300 Foča, Bosnia and Herzegovina; 2 Department of Surgery, University Hospital Foča, Studentska 5, 73300 Foča, Bosnia and Herzegovina; 3 Department of Ophtalmology, University Hospital Foča, Studentska 5, 73300 Foča, Bosnia and Herzegovina; 4 Department of Epidemiology, Faculty of Medicine, University of Pristina Temporarily Seated in Kosovska Mitrovica, Henri Dunant, 38220 Kosovska Mitrovica, Serbia

**Keywords:** outpatient antibiotic consumption, COVID-19, antimicrobial management, mortality

## Abstract

The pandemic of COVID-19 has brought many changes in health care systems at all levels of health care. The increase in the number of cases of COVID-19 has led to overuse and misuse of antibiotics.The aim of this study was to compare the consumption of antibiotics for systemic use in outpatients in the Republic of Srpska (RS), before and during the first year of the COVID-19 pandemic, as well as the association between antibiotic consumption and the rate of incidence and mortality of COVID-19. The total consumption of the antibiotics for systemic use (J01) in outpatients in the Republic of Srpska during 2019 was 19.40 DDD/TID, with an increase to 30.80 DDD/TID in 2020.Significantly higher use of penicillin (10.58 ± 11.01 DDD/TID in 2019 vs. 17.10 ± 13.63 DDD/TID in 2020), cephalosporins (2.68 ± 1.90 DDD/TID in 2019 vs. 5.93 ± 2.77 DDD/TID in 2020) and macrolides (2.14 ± 2.22 DDD/TID in 2019 vs. 3.40 ± 3.44 DDD/TID in 2020) was observed during the pandemic period. It is necessary to improve the prescribing practice of antibiotics at the primary health care level, public awareness about rational use of antibiotics, as well as the current antibiotic stewardship programs and control their implementation.

## 1. Introduction

A case series of viral pneumonia of unknown origin was reported during December 2019 in Wuhan, Hubei, China. In the first reports, the World Health Organization (WHO) associated the occurrence of the disease with the consumption of wildlife meat from the local wet market [1]. However, the new disease promptly spread worldwide, and a pandemic was enunciated on 11 March 2020 [2,3]. Sequencing the full genome from patient samples identified the SARS-CoV-2 virus, the causative agent of the new COVID-19 disease. Clinical manifestations of COVID-19 disease range from asymptomatic or mild forms, with commonsymptoms including fever, cough, sore throat, nasal congestion, fatigue or myalgia, shortness of breath, loss of sense of taste and smell, to severe forms with pneumonia, severe acute respiratory syndrome (ARDS), and multiorgan dysfunctionwith fatal outcomes [4,5].

The COVID-19 pandemic has had extensiveconsequences for all aspects of society, especially disrupting the health systems of even the wealthiest countries [6,7]. The burden on the health system has mostly affected transitional countries, leading to fatal consequences for people’s health and lives. Thus, Bosnia and Herzegovina took the infamous third place in the world according to mortality rate from COVID-19 [8]. So far, there is no detailed analysis of the problems that caused such a high mortality rate. Bosnia and Hercegovina consists of three autonomy entities: the Federation of Bosnia and Herzegovina (FBiH), the Republic of Srpska (RS) and the self-governing District of Brcko (DB) with their own health systems [9]. In order to ensure adequate access to health care and preservehospital capacity for severe cases, the largest number of COVID-19 patients in the RS were referred and treatedat the primary level of health care in specially organized COVID-19 health stations (ambulanta) [10,11].

Unjustified use of antibiotics is a new globalphenomena in the treatment of COVID-19 patients [12,13]. The increase in the consumption of antibiotics is justified by the frequent complications of COVID-19 with bacterial infections that lead to an increase in morbidity and mortality [14,15,16]. On average, 75% of COVID-19 cases worldwide were treated with antibiotics, while only 18% of patients who received antibiotics had secondary bacterial infections. On average, about half of the COVID-19 patients who received antibiotics were neither severely nor critically ill [14]. The increased consumption of reserve antibiotics in hospitals during the COVID-19 pandemic is mainly caused by the increased number of severely ill patients with a lack of antibiotic stewardship programs [15,16].At the primary health care level, a difference in the consumption of antibiotics was registered between individual European countries [10,11]. Although an increase in the consumption of antibiotics was recorded in most countries, there were European countries with opposite results, paradoxically to the pandemic situation and a large number of COVID-19 cases [10,11]. Even before the pandemic, it was recognized that the misuse and overuse of antibiotics contributes to the development of multidrug-resistant organisms and antimicrobial resistance (AMR).

To prevent thedevelopment of antimicrobial resistance, the WHO developed the AWaRe classification of antibiotics classifies key antibiotics into three groups: Access, Watch and Reserve [17]. Most of the antibiotics on the WHO reserve list are not available in RS.Activities to improve the rational use of antibiotics in hospitals of the Republic of Srpska include clinical guidelines with diagnostic and therapeutic principles for inpatient treatment. The list of reserve antibiotics is determined only at the hospital level and includes some of the 3rd generationcephalosporins(ceftazidime, cefotaxime), 4th generation cephalosporins (cefepime), carbapenems (imipenem, meropenem, ertapenem), glykopeptides (vancomycin, teicoplanin), aminoglycosides (amikacin), quinolone (ciprofloxacin, norfloxacin, levofloxacin, moxiflokacin) colistin and linezolid. At the primary health care level, the activity of improving the rational use of antibiotics implies the possibility of issuing antibiotics only on a doctor’s prescription with full, partial or no compensation for outpatients and with established penalties for misuse [18].

The aim of this study was to compare the consumption of antibioticsfor systemic use in outpatients in the Republic of Srpska (RS), before and during the first year of the COVID-19 pandemic. Also, the association of antibiotic consumption with incidence and mortality rates from COVID-19 during the first year of the COVID-19 pandemic was examined.

## 2. Materials and Methods

### 2.1. Design and Data Collection

For this descriptive ecological study, data about antibiotic consumption in outpatients were obtained from the Public Health Institute of the Republic of Srpska (PHI) [19]. Pharmacies regularly submit PHI reports on all released and sold antibiotics prescribed in the outpatient departments. Antibiotics in RS pharmacies can only be dispensed with a prescription from a family doctor at the primary level of health care. Depending on the geographic location, distribution by municipality and their gravitation towards larger regional hospitals, the pharmacies that reported antibiotic consumption were grouped in six regions of the Republic of Srpska (Prijedor, Banja Luka, Doboj, Bijeljina, East Sarajevo and Trebinje). Data on confirmed COVID-19 cases and COVID-19 deaths were obtained from the Public Health Institute of the Republic of Srpska. The data was extracted in two time periods, during 2019, i.e., before COVID-19 pandemic, and during 2020, i.e., during the first year of the pandemic. The first period includes data from 1January to 31 December 2019, and the second period from 1January to 31 December 2020.

The study included all parenteral and oral outpatient antibiotics used during the defined period with overall consumption greater than 0.015 DDD/TID. Antibiotics with a small impact on total consumption (consumption less than 0.015 DDD/TID) were excluded from the study (benzylpenicillin sodium/benzylpenicillin procaine, fosfomycin, pipemidic acid). Antibiotics whose use is allowed only in hospital settings were excluded from the study. For the purposes of the study, antibiotics that were dispensed to outpatients during defined periods were divided into six groups based on anatomical therapeutic chemical (ATC) classification system, with code J01 representing antibacterial for systemic use [20] (Table 1).

### 2.2. Outcome Assessment

The following outcomes were calculated for antibiotic consumption: (1) defined daily dose per thousand inhabitants (DDD/TID) was calculated as the total DDD of antibiotics dispensed in all open-type pharmacies during one calendar year divided by estimated mid-year population of the Republic of Srpska, multiplied by 1000 (2); the ratio DID/TID 2020/DID/TID 2019 was calculated as antibiotic consumption expressed as DDD/TID in 2020 divided by antibiotic consumption expressed as DDD/TID in 2019 [21,22].

The incidence and mortality rates of COVID-19 in the Republic of Srpska was calculatedusing the World Health Organization definitions. Crude incidence and mortality rates were calculated per 100,000 inhabitants. The COVID-19 incidence rate in 2020 was calculated using the formula: the number of confirmed cases of COVID-19 in 2020 divided by the estimated mid-year population of the Republic of Srpska, multiplied by 100,000. The COVID-19 mortality rate in 2020 was calculated using this formula: the number of deathsfrom COVID-19 in 2020 divided by the estimated mid-year population of the Republic of Srpska, multiplied by 100,000. The estimated mid-year population of the Republic of Srpska of 1,136,274 was obtained from the website of Republic Institute of Statistics—Republika Srpska [22].

### 2.3. Statistical Analysis

The methods of descriptive and analytical statistics were used for data description and analysis. Among the methods of descriptive statistics, measures of central tendency and measures of variability were used, namely: arithmetic mean with standard deviation. For the univariate analyses of possible differences in DDD/TID between two time periods the parametric tests the paired-samples *t*-test and nonparametric Wilcoxon test were used. The correlation was done with the help of Spearman’scorrelation coefficient.Overall use of antibiotics was used for the correlation analysis.The usual value of *p* < 0.05 was taken as the level of statistical significance of differences. All statistical analyses were performed using IBM SPSS Statistics Software version 24.0 for Windows (IBM Corp., Armonk, NY, USA).

## 3. Results

**Figure 1:**
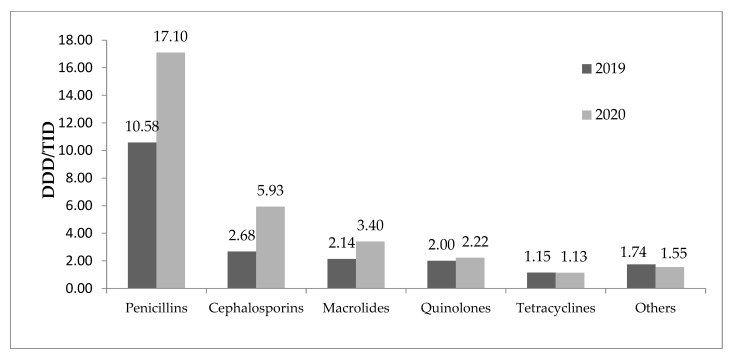
Consumption of antibiotics for systemic use before and during COVID-19 pandemic. DDD/TID—defineddaily dose per 1000 inhabitants.

The total consumption of the antibiotics for systemic use (J01) in outpatients in the Republic of Srpska during 2019 was 19.40 DDD/TID, with an increase to 30.80 DDD/TID in 2020. Significantly higher use of penicillin (10.58 ± 11.01 DDD/TID in 2019 vs. 17.10 ± 13.63 DDD/TID in 2020, *p* < 0.001), cephalosporins (2.68 ± 1.90 DDD/TID in 2019 vs. 5.93 ± 2.77 DDD/TID in 2020, *p* < 0.001) and macrolides (2.14 ± 2.22 DDD/TID in 2019 vs. 3.40 ± 3.44 DDD/TID in 2020, *p* = 0.001) was observed during the pandemic period compared to the period before the COVID-19 pandemic (Table 2). The ratio of penicillin use between 2019 and 2020 was 1.61, the ratio for macrolides was 1.59, while for cephalosporins it was 2.22. The consumption of quinolones, tetracyclines and other antibiotics of J01 group did not change significantly in the studied period (the ratios DDD/TID 2020 and DDD/TID 2019 were 1.11, 0.98 and 0.89, respectively) (Table 2 and Figure 1).

**Figure 2:**
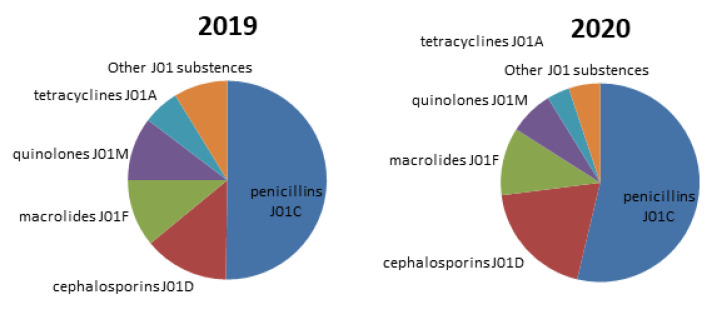
Distribution of consumption of different J01 groups of antibacterialsin 2019 and in 2020.

Regarding the distribution of antibiotic consumption among different J01 groups it was observed that penicillins were the most favorable antibiotics in 2019 and 2020 too. A slight increase in cefalosporinsconsumption was observed in 2020 compare to 2019 (Figure 2).

**Figure 3:**
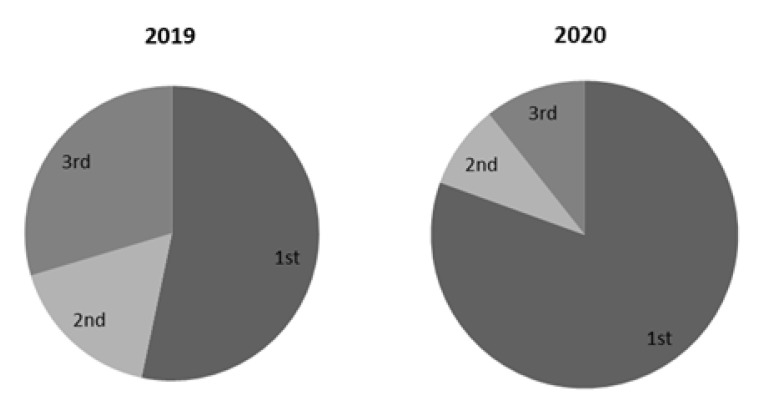
Distribution of different generation of cefalosporins consumption in 2019 and 2020.

In the group of penicillin antibiotics, a significantly higher use of amoxicillin was observed in the period during the first year of the pandemic compared to the period before the pandemic (*p* ≤ 0.001), while a significant difference in the use of ampicillin and amoxicilline/clavunic acid was not observed. Before the pandemic, the use of amoxicillin was 6.60 ± 6.71 DDD/TID, but during the pandemic it increased to 13.21 ± 3.78 DDD/TID. When it comes to cefalosporins, the biggest change was observed in the consumption of cephalexin, i.e., 1st-generation cephalosporins (from 1.60 ± 1.18 before the pandemic to 4.77 ± 2.71 DDD/TID, *p* = 0.009 in the first year of COVID-19 pandemic). The consumption of 2nd generation of cephalosporins decreased, but not significantly, while the consumption of 3rd generation of cephalosporins, specially cefixime, significantly increased in 2020 (the consumption of cefixime increased from 0.24 ± 0.16 to 0.38 ± 0.25 DDD/TID, *p* = 0.032). In total, a significant increase in the consumption of 1st generation of cephalosporins compared to 2nd and 3rd generation of cephalosporins was observed (Figure 3). In the group of macrolides, a significant increase in the use of azithromycin was observed in the first year of the COVID-19 pandemic compared to the period before the pandemic (from 1.27 ± 1.18 to 2.73 ± 2.81 DDD/TID, *p* = 0.007), as well as a decrease in the use of erythromycin from 0.34 ± 0.40 to 0.19 ± 0.13 (*p* = 0.042), while there was no difference in the use of clarithromycin and roxythromycin (Table 2).

By analyzing the use of antibiotic groups by region, it was observed that during the pandemic the use of macrolides (from 6.78 ± 1.44 to 10.80 ± 3.45 DDD/TID, *p* = 0.002) and cephalosporins (from 5.97 ± 1.72 to 7.06 ± 1.87 DDD/TID, *p* = 0.032) increased significantly in Prijedor. The consumption of other antibiotics decreased from 7.69 ± 0.98 to 4.36 ± 0.49 DDD/TID, *p* = 0.001, while no change in penicillin use was observed in this region. In Banja Luka, during the pandemic, there was a significant increase in the use of penicillins (from 8.64 ± 2.15 to 30.84 ± 10.13 DDD/TID, *p* < 0.001), cephalosporins (from 2.02 ± 0.43 to 9.21 ± 4.08 DDD/TID, *p* = 0.003) and macrolides use (from 1.95 ± 0.48 to 2.86 ± 0.91 DDD/TID, *p* = 0.023). The use of macrolides increased significantly in Doboj from 1.07 ± 0.26 to 1.41 ± 0.46, *p* = 0.048, as well as in Bijeljina (from 1..44 ± 0.28 to 2.32 ± 0.73, *p* = 0.009), East Sarajevo (from 1.25 ± 0.23 to 2.80 ± 0.78, *p* < 0.045) and in Trebinje (from 1.29 ± 0.29 to 3.19 ± 1.09, *p* = 0.005), while the consumption of other antibiotics significantly decreased from 12.03 ± 2.61 to 3.55 ± 0.54, *p* = 0.004. Changes in other groups of antibiotics in these regions were not observed. In addition, the index of macrolide use was higher in all regions compared to other antibiotic groups, except in Banja Luka, where the penicillins ratio was the highest (Table 3).

The crude incidence rate of COVID-19 in the Republic of Srpska during 2020 was 3272.58 per 100,000 inhabitants, while the crude mortality rate was 156.91 per 100,000. Table 4 shows the 2020 incidence and mortality rates of COVID-19 by regions.

A strong positive correlation was observed between the consumption of antibiotics among outpatients in the Republic of Srpska during 2020 and crud COVID-19 incidence rate (r = 0.992; *p* < 0.001), as well as crude COVID-19 mortality rate (r = 0.999; *p* < 0.001). This means that higher COVID-19 incidence and mortality rates were followed by significantly higher consumption of antibiotics among outpatients (Table 5).

## 4. Discussion

The increase in outpatients’ consumption of penicillins, cephalosporins and macrolides was registered in the RS during COVID-19 pandemic comparing to the period before the pandemic. Farther analysis showed there are serious differences in the consumption of these groups of antibiotics between different regions of the RS, which implies significant differences in the functioning of primary health care setting in these regions. Penicillins consumption in the entire RS has increased, but the largest increase was recorded in the Banja Luka region, while the changes in other regions were not significant. An increase in the total consumption of cephalosporins in the RS was also registered, with a significant increase in the Banja Luka region, but not in other regions.

Comparing different generations of cephalosporins, the first generation of cephalosporins had the largest contribution to the total consumption of cephalosporins, and in this group the largest increase in consumption was registered during the pandemic compared to the period before the pandemic. This is not consistent with the consumption of cephalosporins in the European region, where the highest consumption is recorded for second-generation cephalosporins [23]. A probable explanation for such a large outpatient consumption of first-generation cephalosporins is the fact that cephalexin is the only cephalosporin that can be obtained with prescription without extra compensation in the RS, or more precisely, the low living standard compared to the high prices of other cephalosporins. Contrary to the results of our study, in 2020 there was a decrease in consumption of first-generation cephalosporins and amoxicillin in Brazil, which was explained by a decrease in all respiratory infections except for COVID-19 [24]. The consumption of macrolides was significantly higher in 2020 compared to 2019 in all regions of the RS, which was also registered in all neighboring countries and globally [25]. The increased consumption of azithromycin is explained by its previously proven immunomodulatory and antiviral effect, which along with antibacterial effect protects against the worsening of viral infections [26,27]. However, some clinical studies have shown that the use of azithromycin did not have any positive effect in the treatment of patients with COVID-19 [28,29]. Unnecessary use of azithromycin may lead to increased resistance rates of respiratory pathogens in addition to a false sense of protection against COVID-19. In the newer version of the guidelines for treatment of patients with COVID-19 strong recommendations were made against the use of azithromycin alone or azithromycin in combination with hydroxychloroquine, and colchicine [29].

Differences in antibiotic consumption between regions may be due to differences in the organization of primary care settings, as well as regional socioeconomic differences. The cause of the increased prescription and consumption of antibiotics is online or telephone consultations, counseling and treatment of COVID-19 cases, especially at the beginning of the pandemic due to the reorganization of the health system. Panic among patients and doctors due to the large number of infected, insufficient knowledge of the clinical course of the disease, the lack of antiviral and immunomodulatory drugs, as well as the low trust of the population in vaccines influenced the excessive use of antibiotics in the observed period [30]. Also, it is possible that the increased sale of antibiotics without a prescription, although prohibited, has contributed to the overuse of antibiotics, which will certainly affect the rate of antimicrobial resistance, but this is beyond the scope of this paper [31].

The consumption of antibiotics in certain European countries decreased during 2020, which is explained by strict adherence to the anti-epidemic measures (masks, physical distance, enhanced hygiene measures), reduced number of visits to primary health care setting and reduced number of non-COVID-19 acute respiratory infections [12,23,32]. One of the reasons for the reduced use of antibiotics in some European countries may be caused by the existence of previously developed antibiotic stewardship programs [33]. However, according to the ESAC Net database overall consumption of antibiotics during COVID-19 pandemic was 30.80 DDD/TID compared to 19.40 DDD/TID before pandemic [12,23,32]. According to the same source, the largest increase in antibiotic consumption in 2020 compared to 2019 was registered in Bulgaria and Romania, which report higher antibiotic consumption compared to the average of most other European countries [23]. The consumption of antibiotics in the RS can be compared with the consumption in Bulgaria, Romania and Greece, and is significantly higher than the consumption in developed European countries such as Sweden, the Netherlands and Austria [23].

Analysis of the consumption of individual antibiotics indicated that the most commonly prescribed antibiotic was amoxicillin. In the group of cephalosporins, a significant increase in the use of cephalexin and cefixime was found. Among macrolides, the increased consumption of azithromycin and decreased consumption of erythromycin was registered. The decreased use of erythromycin is probably the consequence of the change in drug selection influenced by early recommendations related to the possibly positive effectsa of azithromycin on COVID-19, as well as its better tolerability and pharmacokinetics when compared to erythromycin [26]. There is evidence of the significant correlation between previous exposure to antibiotics and increased severity of COVID-19 disease. It is considered that previous exposure to antibiotics may lead to the avoidance of the antiviral mechanisms of the immune cells of patients [13].

In our study, a correlation was registered between the consumption of antibiotics and the COVID-19 incidence and mortality rates. In the region of Banja Luka, the highest consumption of antibiotics was registered, which correlates with the highest COVID-19 incidence and mortality rates. The highest incidence of COVID-19 in this region may be connected to the greatest density of population in this region, which puts individuals at greater risk of the transmission and spreading of the infection. As Banja Luka is the capital of the Republic of Srpska, this region is highly populated with the largest number of highly educated and employed inhabitants, which, due to the intensity of contact, is associated with the registered high rate of COVID-19 and higher antibiotic prescriptions. Indirectly, the results of this study lead to the conclusion that the increased consumption of antibiotics did not reduce the COVID-19 mortality rate in RS. Incorrect use of antibiotics increases antibiotic resistance, which can affect the further increase in mortality of COVID-19 patients, especially due to bacterial superinfections caused by multi-resistant bacteria [34].

## 5. Conclusions

It is necessary to take certain measures to improve the practice of prescribing antibiotics at the primary health care level and to develop the quaternary prevention in the sense of protection against excessive medicalization. In order to educate healthcare professionals and to raise public awareness of the importance of rational antibiotic use, promotional and educational programs need to be created, and existing ones improved. It is necessary to improve the current antibiotic stewardship programs and control their implementation.

## 6. Limitations

The lack of information on indications for prescribed antibiotics limited us from more precisely classifying antibiotics according to the AMT code J01 classification. The second limitation is ecological fallacy, which means that conclusion on a level of a group cannot be applied to a separate case and therefore no conclusions about causality can be drawn. Furthermore, the shortcoming of the study is that we did not analyze the consumption of antibiotics by waves of COVID-19 during 2020, and therefore we could not monitor whether the increased in antibiotics consumption follows increases in the number of COVID-19 cases. The study did not include dataon the consumption of antibiotics at the secondary and tertiary level, but only at the level of primary health care, so we do not have a complete picture of the consumption of antibiotics at all levels of health care. The strength of this study was the representative sample size. The data for this study were obtained at the state level and can therefore be used to plan futher health policy development, allocate health resources and improve antibiotic stewardship programs at the national level. A strength of this study was the representative sample size. The data for this study were obtained at the national level and can therefore be used to plan further health policy development, allocate health resources and improve protocols for the rational use of antibiotics at the national level.

## Figures and Tables

**Table 1:** Groups of antibiotics used in outpatients in RS during 2019 and 2020.

Penicillins J01C	Cephalosporins J01D	Macrolides J01F	Quinolones J01M	Tetracyclines J01A	Other
Phenoxymeth * J01CE02	cephalexin J01DB01	Erythromycin J01FA01	ciprofloxacin J01MA02	doxycycline J01AA02	TMS *** J01EE01
ampicillin J01CA01	cefuroxime J01DC02	Roxithromycin J01FA06	nofloxacin J01MA06		clindamycin J01FF01
Amoxicillin J01CA04	cefaclor J01DC04	clarithromycin J01FA09	levofloxacin J01MA12		linkomycin J01FF02
amox/clav ** J01CR02	ceftriaxone J01DD04	Azithromycin J01FA10	moxifloxacin J01MA14		gentamicin J01GB03
	cefixim J01DD08				metronidazole J01XD01
	cefpodoxim J01DD13				Nitrofurantoin J01XE01

* phenoxymethylpenicillin; ** amoxicillin/clavulanic acid, *** trimethoprim/sulfamethoxasole.

**Table 2:** Defined daily dose per 1000 inhabitants and the DID/TID ratio in 2019 and 2020 of consumption of antibiotics for systemic use (J01) before (2019) and during the pandemic period (2020) in RS.

Groups of Antibiotics	INN	ATC	DDD/TID				Ratio	*p*
			2019		2020			
			Mean	SD	Mean	SD		
Penicillins	Phenoxymet	J01CEO2	0.84	0.33	0.55	0.22	0.66	0.211 **
	Amoxicillin	J01CA04	6.60	6.71	13.21	3.78	2.00	<0.001 **
	Ampicillin po	J01CA01	0.14	0.35	0.09	0.14	0.61	0.881 **
	Ampicillin pe		0.01	0.20	0.01	0.11	0.90	0.921 *
	Amox/clav po	J01CR02	2.95	4.13	2.85	0.71	0.97	0.532 **
	Amox/clav pe		0.04	0.04	0.39	0.20	9.28	0.198 **
TOTAL			10.58	11.01	17.10	13.63	1.61	<0.001 **
Cephalosporins	Cefalexin	JO1DB01	1.60	1.18	4.77	2.71	2.98	0.009 *
SUM 1st generation			1.60	1.18	4.77	2.71	2.98	0.009 *
	Cefuroxim po	J01DC02	0.57	0.22	0.50	0.20	0.87	0.257 *
	Cefuroxim pe		0.01	0.02	0.01	0.01	0.76	0.890 **
	Cefaclor	J01DC04	0.03	0.01	0.02	0.01	0.51	0.711 **
SUM 2nd generation			0.61	0.25	0.52	0.21	0.85	0.562 *
	Cefixim po	J01DD08	0.24	0.16	0.38	0.25	1.60	0.032 *
	Cefpodoxim	J01DD13	0.04	0.05	0.05	0.08	1.21	0.921 **
	Ceftriaxon	J01DD04	0.18	1.10	0.20	1.45	1.09	0.111 **
SUM 3rd generation			0.46	1.15	0.63	1.48	1.37	0.211 **
TOTAL			2.68	1.90	5.93	2.77	2.22	<0.001 **
Macrolides	Azythromycin po	J01FA10	1.27	1.18	2.73	2.81	2.15	0.007 **
	Azithromycin pe		0.01	0.02	0.06	0.11	5.27	0.332 **
	Eryithromycin	J01FA01	0.34	0.40	0.19	0.13	0.57	0.042 **
	Clarithromycin po	J01FA09	0.49	0.64	0.40	0.41	0.82	0.092 **
	Clarithromycinepo		0.00	0.01	0.01	0.01	4.71	0.821 **
	Roxithromycine	J01FA06	0.02	0.04	0.01	0.01	0.44	0.623 **
TOTAL			2.14	2.22	3.40	3.44	1.59	0.001 **
Quinolones	Ciprofloxacin po	J01MA02	1.43	0.89	1.29	0.74	0.90	0.192 **
	Ciprofloxacin pe		0.02	0.02	0.03	0.01	1.58	0.581 **
	Levofloxacin	J01MA12	0.32	0.22	0.65	0.29	2.02	0.098 **
	Moxifloxacin	J01MA14	0.03	0.04	0.06	0.09	2.07	0.299 **
	Moxifloxacin pe		0.01	0.01	0.02	0.03	3.07	0.760 *
	Norfloxacin	J01MA06	0.19	1.41	0.17	1.33	0.88	0.812 **
TOTAL			2.00	2.55	2.22	2.46	1.11	0.449 **
Tetracyclines	Doxycycline	J01AA02	1.15	0.58	1.13	1.04	0.98	0.771 **
	TMS	J01EE01	1.04	0.96	0.86	0.49	0.83	0.192 **
	Lincomycin po	J01FF02	0.05	0.18	0.03	0.06	0.53	0.792 **
	Lincomycin pe		0.00	0.01	0.00	0.00	0.58	0.891 **
	Clindamycin po	J01FF01	0.04	0.11	0.04	0.17	1.08	0.992 **
	Clindamycin pe		0.00	0.03	0.01	0.03	1.45	0.875 **
	Gentamicin	J01GB03	0.04	1.13	0.03	1.15	0.79	0.569 **
	Nitrufurantoin	J01XE01	0.15	1.23	0.23	2.00	1.50	0.772 **
	Metronidazol po	J01XD01	0.01	1.15	0.03	1.45	3.84	0.780 **
	Metronidazol pe		0.40	3.18	0.32	0.19	0.78	0.298 **
TOTAL			1.74	0.34	1.55	0.28	0.89	0.662 **

DDD/TID—defined daily dose per 1000 inhabitants; INN—international non-proprietary name; Phenoxymet—phenoxymethylpenicillin; Amox/clav—amoxicillin/clavulanic acid; TMS—trimethoprim/sulfamethoxasole; po–per oral; pe—parenteral; * paired-samples *t*-test; ** Wilcoxon test; SD—standard deviation; *p*—statistical significanc.

**Table 3:** Defined daily dose per 1000 inhabitants for penicillins, cephalosporins and macrolides use in outpatients, before (2019) and during (2020) pandemic period in RS.

Region of the Republic of Srpska	Groups of Antibiotics	DDD/TID				*p*
		2019		2020		
		Mean	SD	Mean	SD	
Prijedor	Penicillins	33.65	13.78	37.99	9.93	0.291 *
	Cephalosporins	5.97	1.72	7.06	1.87	0.039 *
	Macrolides	6.78	1.44	10.80	3.45	0.002 *
	Quinolones	6.95	1.58	7.28	1.49	0.078 **
	Tetracyclines	2.01	0.92	3.05	1.45	0.219 **
	Others	7.69	0.98	4.36	0.49	0.001 **
Banja Luka	Penicillins	8.64	2.15	30.84	10.13	<0.001 *
	Cephalosporins	2.02	0.43	9.21	4.08	0.003 *
	Macrolides	1.95	0.48	2.86	0.91	0.023 *
	Quinolones	1.75	0.39	1.97	0.36	0.562 **
	Tetracyclines	1.19	1.01	0.93	0.62	0.491 **
	Others	4.80	0.64	4.06	0.55	0.319 **
Doboj	Penicillins	7.19	1.70	6.38	1.54	0.078 *
	Cephalosporins	1.38	0.27	1.63	0.30	0.639 *
	Macrolides	1.07	0.26	1.41	0.46	0.048 *
	Quinolones	1.07	0.22	1.04	0.19	0.344 **
	Tetracyclines	0.64	0.21	0.40	0.33	0.827 **
	Others	2.40	0.34	2.67	0.40	0.182 **
Bijeljina	Penicillins	6.76	1.66	10.73	2.75	0.056 *
	Cephalosporins	0.17	0.42	1.82	2.22	0.872 *
	Macrolides	1.44	0.28	2.32	0.73	0.009 *
	Quinolones	1.41	0.33	1.73	0.38	0.412 **
	Tetracyclines	1.42	0.90	1.22	0,88	0.806 **
	Others	5.73	1.30	6.32	1.48	0.002 **
East Sarajevo	Penicillins	5.79	1.05	8.64	2.32	0.276 *
	Cephalosporins	5.22	1.46	3.74	0.67	0.491 *
	Macrolides	1.25	0.23	2.80	0.78	0.045 *
	Quinolones	0.94	0.19	1.13	0.20	0.464 **
	Tetracyclines	0.52	0.14	0.69	0.51	0.374 **
	Others	7.50	1.22	5.62	1.14	0.091 **
Trebinje	Penicillins	5.61	0.99	8.29	2.22	0.322 *
	Cephalosporins	3.21	0.64	6.79	2.22	0.079 *
	Macrolides	1.29	0.29	3.09	1.09	0.005 *
	Quinolones	0.64	0.17	0.88	0.20	0.093 **
	Tetracyclines	0.68	0.48	0.89	0.52	0.081 **
	Others	12.03	2.61	3.55	0.54	0.004 **

DDD/TID—defined daily dose per 1000 inhabitants; * paired-samples *t*-test; ** Wilcoxon test; SD—standard deviation; *p*—statistical significance.

**Table 4:** Crude incidence and mortality rates of COVID-19 in 2020 in the RS.

Region of the Republic of Srpska	Incidence Rate	Mortality Rate
Prijedor	1467.80	145.39
Banja Luka	4248.47	183.45
Doboj	1947.16	159.90
Bijeljina	3019.22	109.39
Istočno Sarajevo	3763.21	160.90
Trebinje	4318.14	171.51

**Table 5:** Correlation between consumption of antibiotics among outpatients and crude COVID-19 incidence and mortality rate in 2020 in the Repbublic of Srpska.

Consumption of Antibiotics in Regions in the Republic of Srpska	Incidence Rate r (*p*)	Mortality Rate r (*p*)
Consumption of antibiotics (DDD/TID) in 2020	0.992 (<0.001)	−0.998 (<0.001)

## Data Availability

The data underlying the results presented in the study are available from: office-mf@ues.rs.ba.
